# Association Between Medicaid Prescription Drug Limits and Access to Medications and Health Care Use Among Young Adults With Disabilities

**DOI:** 10.1001/jamahealthforum.2021.1048

**Published:** 2021-06-17

**Authors:** Caroline K. Geiger, Jessica L. Cohen, Benjamin D. Sommers

**Affiliations:** 1 Harvard University, Interfaculty Initiative in Health Policy, Cambridge, Massachusetts; 2 Genentech, Inc, South San Francisco, California; 3 Department of Global Health and Population, Harvard T. H. Chan School of Public Health, Boston, Massachusetts; 4 Department of Health Policy and Management, Harvard T. H. Chan School of Public Health, Boston, Massachusetts; 5 Harvard Medical School/Brigham & Women’s Hospital, Boston, Massachusetts

## Abstract

**Importance:**

Prescription drugs are necessary for managing complex physical and mental health conditions for more than 10 million Medicaid beneficiaries with disabilities. However, some state Medicaid programs limit the number of prescription drugs that beneficiaries can obtain monthly, which may decrease access to essential medications.

**Objective:**

To examine the association between exposure to the 3-drug limit at age 21 years in Arkansas and Texas and prescription drug and health care use among beneficiaries with disabilities enrolled in Medicaid.

**Design, Setting, and Participants:**

In this cohort study of 28 046 young adults with disabilities, difference-in-differences analysis was performed using Medicaid Analytic eXtract claims data from January 1, 2007, to December 31, 2012. Analyses were completed December 1, 2020. The analyses included Medicaid beneficiaries with disabilities in Arkansas and Texas (ie, drug cap states) or 15 comparison states without drug cap policies who became age 21 years during the study period and were continuously enrolled in fee-for-service Medicaid in the year before and after that point.

**Exposures:**

Exposure to the 3-drug prescription limit at age 21 years in 2 drug cap states.

**Main Outcomes and Measures:**

Monthly total prescriptions and prescriptions for drugs to treat mental health conditions, total prescription drug spending, and inpatient and emergency department visits and spending in the 12 months before and after becoming age 21 years.

**Results:**

Among 28 046 young adults with disabilities, 8214 (29.3%) resided in drug cap states and were subject to the 3-drug limit at age 21 years. Most individuals were male (drug cap states: 61.4%, comparison states: 60.6%), and the minority were White individuals (drug cap states: 36.7%, comparison states: 49.4%). More than one-half of individuals with disabilities were diagnosed with a mental health condition before age 21 years (drug cap states: 57.0%, comparison states: 60.0%). In the year before the analyzed individuals became aged 21 years, the mean (SD) number of prescriptions per beneficiary per month was 1.58 (2.16) in drug cap states vs 1.82 (1.91) in comparison states. The drug cap policy was associated with 19.6% (95% CI, −21.3% to −17.8%; *P* < .001) fewer monthly prescriptions and 16.5% (95% CI, −21.9% to −10.8%; *P* < .001) fewer prescriptions for drugs for mental health conditions but was not associated with total prescription drug spending. The drug cap policy was associated with 13.6% (95% CI, 1.9% to 26.6%; *P* = .02) more inpatient admissions.

**Conclusions and Relevance:**

In this cohort study of young adults with disabilities, drug cap policies were associated with lower rates of access to important medications and higher rates of hospitalization among individuals in states with drug cap policies vs those without these policies.

## Key Points

**Question:**

Are policies that cap monthly prescriptions in Medicaid associated with access to medication and health care use among young adults with disabilities in Arkansas and Texas?

**Findings:**

In this cohort study using difference-in-differences analysis of 28 046 young adults with disabilities, including 8214 in states with a 3-drug limit at age 21 years, the 3-drug limit was associated with lower monthly prescriptions for medications used to treat mental health conditions and higher inpatient admissions among all individuals with disabilities in states with the drug cap policy compared with those in states without this policy.

**Meaning:**

In this study, state drug cap policies in Medicaid were associated with lower access to medications and higher use of inpatient care.

## Teaser

This cohort study examines changes in the rates of access to medications and health care by individuals with disabilities in US states that have instituted caps to prescription drugs available through Medicaid.

## Introduction

As of 2018, more than 10 million individuals enrolled in state Medicaid programs qualified for coverage due to a disability, including physical or mental health conditions, intellectual or developmental disabilities, or functional limitations.^1^ Most beneficiaries with disabilities have multiple chronic conditions, and nearly half have a serious mental illness.^2^ Prescription drugs are often necessary to manage physical and mental conditions for Medicaid beneficiaries with disabilities.^3^ However, many state Medicaid programs use drug cap policies that aim to control costs by limiting the number of monthly prescriptions a patient can fill.^4,5^

As of 2019, 13 states had adopted Medicaid drug caps, with limits as low as 3 drugs per month in Texas and Arkansas.^5,6^ Previous research found that the 3-drug limit implemented in New Hampshire in 1981 was associated with decreases in both essential and nonessential drugs, as well as increases in acute mental health services and admissions to nursing homes.^7,8,9,10^ The implementation of more recent drug cap policies has been associated with state-level decreases in total prescriptions among all beneficiaries.^5^ Conversely, the removal of drug cap policies for dual Medicare-Medicaid–eligible beneficiaries in 2006 has been associated with increases in access to important medications.^11,12,13^

Little is known about the influence of drug cap policies on prescriptions and spending among young, nondual Medicaid beneficiaries with disabilities who may need multiple medications to manage complex health needs.^3,10^ Discontinuation of and nonadherence to necessary medications could lead to increases in hospitalizations and medical costs as well as lower quality of life, but drug caps have not been evaluated in this particular population.^14,15,16,17,18^ To our knowledge, no recent studies have examined drug caps’ potential associations with other types of health care use, including inpatient and emergency care.

This study evaluated the association between the 3-drug limit in Arkansas and Texas, which takes effect when Medicaid beneficiaries become age 21 years, and the total number and types of prescription drugs used and inpatient and emergency department visits among young adults with disabilities. We also examined the association separately for individuals with a serious mental illness who may be at the highest risk for adverse events owing to reduced access to medications.

## Methods

### Study Design

This cohort study used the natural experiment created by the imposition of the 3-drug limit on Medicaid beneficiaries starting at age 21 years. Using difference-in-differences methods, we compared differences in the use of prescription drugs and health care services for individuals with disabilities in the 12 months before vs after age 21 years in states with a drug cap policy vs a set of comparison states with no drug cap policy. We focused on Arkansas and Texas because these states had the most restrictive drug cap policy (ie, lowest monthly limit on prescriptions) and did not have any other drug rationing policies (eg, copayments) that also went into effect at age 21 years. The 3-drug limit in both states excludes family planning and smoking cessation prescriptions, and Texas also excludes insulin syringes. In Arkansas, the state Medicaid program will consider individual requests for an extension of the limit to 6 drugs per month for those deemed to be at risk of institutionalization.^19,20^

### Data Source

The primary data source for this study was the Medicaid Analytic eXtract (MAX) administrative claims data (January 1, 2007, to December 31, 2012). Data analysis was completed December 1, 2020. The MAX data include all medical and prescription drug claims as well as details on monthly enrollment for all Medicaid beneficiaries. This study was deemed exempt by the Harvard University Institutional Review Board because data were deidentified. This study followed the Strengthening the Reporting of Observational Studies in Epidemiology (STROBE) reporting guideline for cohort studies.

This study also used data from the National Library of Medicine to classify prescription drugs based on their National Drug Code into classes using the Anatomical Therapeutic Chemical classification system. Using the National Library of Medicine’s RxNorm and RxClass, each National Drug Code in the MAX data was mapped to 1 or more Anatomical Therapeutic Chemical class.^21^

### Sample

This study included all young adults with disabilities who were enrolled in full-benefit fee-for-service Medicaid. All individuals enrolled in Medicaid owing to a disability when younger than 21 years who became 21 years during the study period (January 1, 2007-December 31, 2012) were included in the sample. Beneficiaries were required to have 12 months of continuous Medicaid enrollment in the same state both before and after becoming 21 years. Individuals were excluded if they were pregnant, eligible for Medicaid as a foster child, or in a long-term care facility in the 12 months before becoming age 21 years or were dually enrolled in Medicaid and Medicare at any time in the 12 months before or after turning age 21 based on enrollment records in the MAX data because they were not subject to the drug cap.

All individuals with disabilities in Arkansas and Texas who were older than 21 years were included in the treatment group (ie, drug cap states). The comparison group included all individuals residing in a state that did not have any drug cap policy, did not have any other drug-rationing policies go into effect at age 21 years, and did not enroll all beneficiaries with disabilities in comprehensive Medicaid Managed Care. Comparison states included Alaska, Colorado, Connecticut, Florida, Idaho, Indiana, Missouri, Nebraska, New Hampshire, New Jersey, New Mexico, Nevada, Oregon, Washington, and Wisconsin.

Young adults with disabilities were also included in the subgroup of individuals with a serious mental illness if they had at least 2 diagnosis codes for schizophrenia and psychotic disorders (*International Classification of Diseases, Ninth Revision, Clinical Modification* codes 295 or 297) or bipolar disorder (codes 296.0, 296.1, 296.4, 296.5, 296.6, 296.7, 296.8, 296.9, 301.11, or 301.13) at any time before age 21 years.^22^ Individuals diagnosed with these conditions often need multiple prescription medications to manage their conditions and may be most affected by the prescription drug cap limit.^23^

### Outcomes

The primary outcomes of interest were the number of outpatient prescription drugs per month overall and stratified by drug class. A prescription refers to 1 instance of an outpatient prescription medication filled and picked up by the patient. Total monthly prescriptions were calculated for all outpatient drugs as well as all medications used to treat mental health conditions and the subclasses of antipsychotics, anxiolytics, antidepressants, and psychostimulants (eTable 1 in the Supplement). We also analyzed whether an individual had more than 3 prescriptions in each month, total monthly prescription drug spending (including all fee-for-service payments by Medicaid for outpatient prescription drugs), and mean spending per prescription drug in each month. Prescription spending did not include cost sharing, because this information is not available in the MAX database.

Secondary outcomes included measures of health care visits and spending. Visit outcomes included the total number of emergency department visits, any inpatient admission, and total inpatient length of stay in each quarter. The total number of outpatient visits was not included because the coding of these visits was inconsistent across states and over time. Inpatient and emergency department spending included all fee-for-service payments made by Medicaid for each visit-type in each quarter and does not include any cost sharing. Total combined spending on inpatient admissions, emergency department, and prescription drug spending in each quarter was also analyzed. Spending was updated to 2020 US dollars using the medical component of the Consumer Price Index.

### Statistical Analysis

For each outcome, the individual-level regression model included an indicator for living in a drug cap state (Arkansas or Texas) and an indicator for whether the individual was older than 21 years in that month or quarter as well as an interaction between the 2 indicator variables. The coefficient on the interaction term measures the association between the drug cap policy and the outcomes of interest. All regressions were adjusted for individual sex and race/ethnicity as defined in the MAX data (White, Black, Hispanic, or other). We classified Asian, Pacific Islander, and Native American individuals as other race due to small sample sizes. Regressions were also adjusted for whether the individual lived in an urban county (Rural Urban Continuum Codes 1-3) to improve precision. The regressions also included state, month, and year fixed effects. Heteroskedastic-robust standard errors were clustered at the individual level.^24^ Regressions were estimated using a zero-inflated negative binomial model for count outcomes; logistic regressions were used for binary outcomes. Further details on the statistical analyses are in the eMethods in the Supplement.

We conducted several sensitivity analyses. First, to further explore changes in prescribing patterns, we analyzed variations in total days’ supply across all prescriptions and mean days’ supply per prescription per month. To further explore prescription spending, we analyzed total prescriptions stratified by drug cost quartiles and total brand and generic prescriptions. In addition, we calculated clustered standard errors by state for our main analyses. We then tested whether trends in outcomes prior to age 21 years were parallel across drug cap and comparison states because the difference-in-differences study design relies on the assumption of parallel trends. Details on the sensitivity analyses are included in the eMethods in the Supplement. All analyses were implemented using Stata/MP, version 15 (StataCorp LLC). Results were considered statistically significant at 2-tailed unpaired *P* < .05.

## Results

The study sample included a total of 28 046 young Medicaid beneficiaries with disabilities (eTable 2 in the Supplement). Among all individuals, 8214 resided in a drug cap state and were subject to the 3-drug limit at age 21 years. Most individuals in both drug cap and comparison states were male (drug cap: men, 5046 [61.4%]; women, 3168 [38.6%] vs comparison: men, 12 020 [60.6%]; women, 7812 [39.4%]), and individuals in drug cap states were less likely to be White vs those in comparison states (drug cap: 3016 [36.7%] vs comparison: 9800 [49.4%]) (Table 1). In both the drug cap and comparison states, more than one-half of the individuals were diagnosed with a mental health condition before age 21 years (drug cap: 4684 [57.0%] vs comparison: 11 891 [60.0%]). Among all young adults with disabilities, 4135 individuals were included in the subgroup of those with a serious mental illness (drug cap: 1178 [14.3%] vs comparison: 2957 [14.9%]).

**Table 1.** Baseline Characteristics of All Beneficiaries With Disabilities in the Year Before Becoming Age 21 Years^a^

Characteristic	No. (%)			
	All individuals with disabilities		Individuals with disabilities and a serious mental illness	
	Drug cap states	Comparison states	Drug cap states	Comparison states
No.	8214	19 832	1178	2957
Demographic characteristic				
Sex				
Male	5046 (61.4)	12 020 (60.6)	722 (61.3)	1888 (63.8)
Female	3168 (38.6)	7812 (39.4)	456 (38.7)	1069 (36.2)
Race/ethnicity				
White	3016 (36.7)	9800 (49.4)	483 (41.0)	1674 (56.6)
Black	1831 (22.3)	3389 (17.1)	273 (23.2)	495 (16.7)
Hispanic	2088 (25.4)	1729 (8.7)	236 (20.0)	222 (7.5)
Residence in urban county	6075 (74.0)	15 272 (77.0)	869 (73.8)	2250 (76.1)
Diagnoses				
Chronic obstructive pulmonary disease	1294 (15.8)	3149 (15.9)	259 (22.0)	779 (26.3)
Asthma	868 (10.6)	2304 (11.6)	164 (13.9)	597 (20.2)
Diabetes	363 (4.4)	846 (4.3)	95 (8.1)	224 (7.6)
Epilepsy	1103 (13.4)	2472 (12.5)	141 (12.0)	319 (10.8)
Mental health conditions	4684 (57.0)	11 891 (60.0)	1178 (100)	2957 (100)
Attention-deficit/hyperactivity disorder	1039 (12.6)	2659 (13.4)	322 (27.3)	1068 (36.1)
Anxiety	1009 (12.3)	2883 (14.5)	384 (32.6)	1262 (42.7)
Depression	1228 (15.0)	3166 (16.0)	497 (42.2)	1412 (47.8)
Schizophrenia and psychotic disorders	456 (5.6)	1290 (6.5)	427 (36.2)	1198 (40.5)
Bipolar disorder	1109 (13.5)	2755 (13.9)	965 (81.9)	2402 (81.2)
Substance use disorder	235 (2.9)	1031 (5.2)	123 (10.4)	565 (19.1)
Developmental disorders	3150 (38.3)	6916 (34.9)	455 (38.6)	1143 (38.7)
Autism	589 (7.2)	1640 (8.3)	82 (7.0)	251 (8.5)

^a^ Analysis included Medicaid Analytic eXtract claims data (January 1, 2007, to December 31, 2012). The sample of all individuals with disabilities included all Medicaid beneficiaries who were eligible for Medicaid owing to a disability before age 21 years and were continuously enrolled in fee-for-service Medicaid in the year before and after becoming 21 years. The serious mental illness subgroup included all patients with disabilities who were diagnosed with schizophrenia and psychotic disorders or bipolar disorder at any time before their 21st birthday. The drug cap states include all individuals residing in Arkansas and Texas who were eligible for the drug cap policy at age 21 years. The comparison states include all individuals residing in Alaska, Colorado, Connecticut, Florida, Idaho, Indiana, Missouri, Nebraska, New Hampshire, New Jersey, New Mexico, Nevada, Oregon, Washington, and Wisconsin who were not eligible for a drug cap policy at age 21 years. Diagnoses include those made at any time before age 21 years.

### Medication Use

In all young adults with disabilities, the total number of monthly prescriptions increased steadily in the 12 months before age 21 years among individuals in both drug cap (mean [SD], 1.58 [2.16]) and comparison (1.83 [2.61]) states, but a sharp decrease in prescriptions at age 21 years was observed in the drug cap states only (Figure, A; eTable 3 in the Supplement). The drug cap policy was associated with 19.6% (95% CI, −21.3% to 17.8%; *P* < .001) lower monthly total prescriptions and 42.4% (95% CI, −44.6% to −40.0%; *P* < .001) fewer months with more than 3 prescriptions (Table 2).

**Figure.**
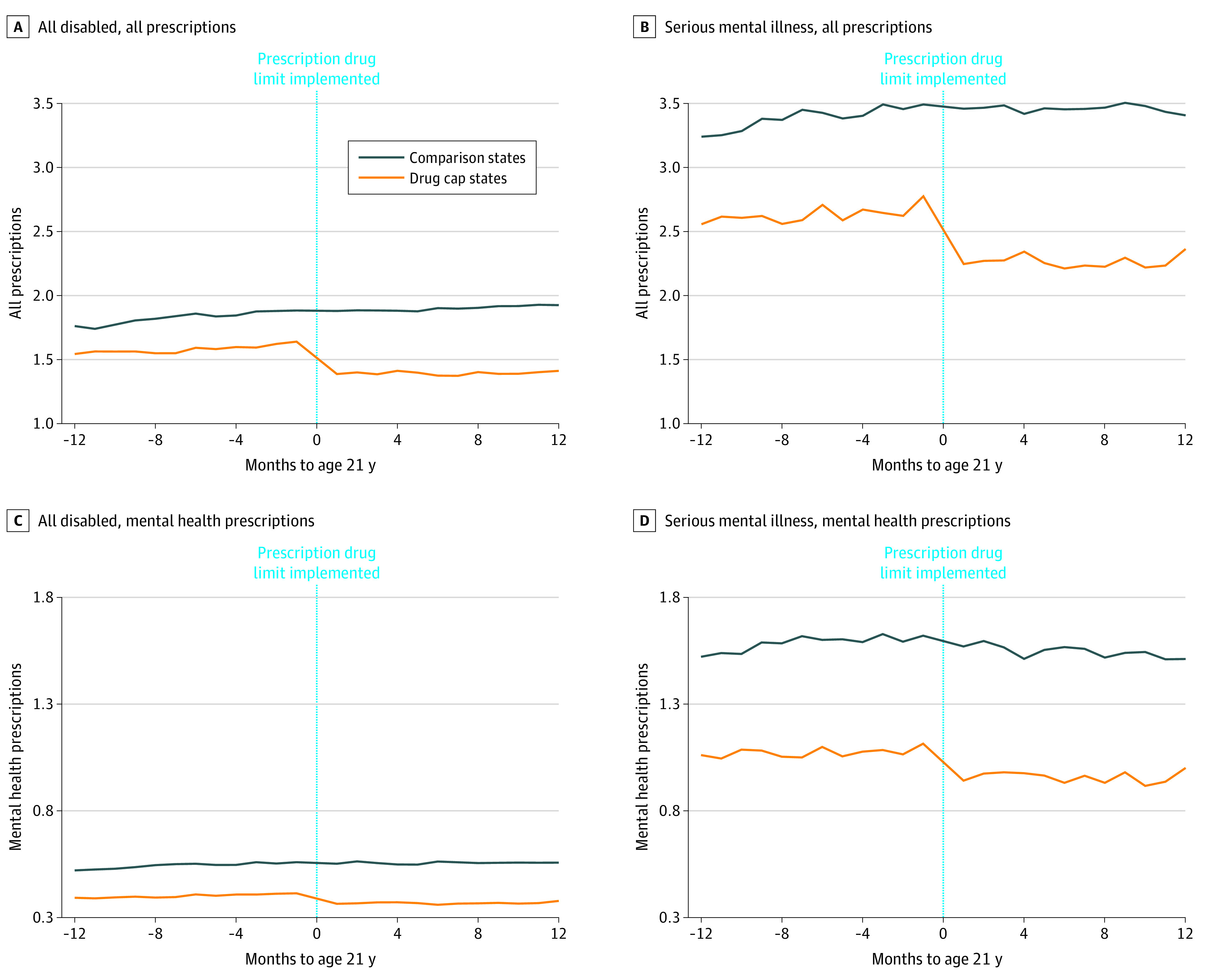
Monthly Prescriptions Before and After Exposure to the Drug Cap Policy at Age 21 Years Data presented are for the mean total prescriptions and mean total prescriptions for drugs used to treat mental health conditions in each month in the year before and after becoming age 21 years for all individuals with disabilities in drug cap states (n = 8214) and comparison states (n = 19 832) as well as the subgroup of individuals with a serious mental illness in drug cap states (n = 1178) and comparison states (n = 2957). The vertical line represents the month of the 21st birthday when individuals were first exposed to the 3-drug limit in drug cap states. The drug cap policy was associated with a significant decrease in total prescriptions and prescriptions for drugs to treat mental health conditions among all individuals with disabilities as well as those with a serious mental illness.

**Table 2.** Changes in Monthly Prescriptions After Exposure to the Drug Cap Policy at Age 21 Years^a^

Prescription drug outcomes	Difference-in-differences estimates					
	All individuals with disabilities (N = 28 046)			Individuals with disabilities and a serious mental illness (n = 4135)		
	Pre–age 21 y in drug cap states, mean (SD)	IRR/OR (95% CI)	*P* value	Pre–age 21 y in drug cap states, mean (SD)	IRR/OR (95% CI)	*P* value
Monthly prescriptions overall						
Total prescriptions	1.58 (2.16)	0.804 (0.787-0.822)	<.001	2.63 (2.65)	0.803 (0.771-0.837)	<.001
>3 Prescriptions per month, %	16.54 (28.59)	0.576 (0.554-0.600)	<.001	29.95 (34.87)	0.535 (0.488-0.586)	<.001
Monthly prescription spending						
Total prescription spending, $	305.92 (1019.98)	0.996 (0.917-1.082)	.93	565.32 (777.01)	0.942 (0.899-0.987)	.01
Total spending per prescription, $	91.24 (412.07)	1.133 (1.038-1.237)	.005	147.64 (160.30)	1.098 (1.040-1.160)	<.001
Monthly prescriptions by ATC class						
All mental health drugs	0.40 (0.78)	0.835 (0.781-0.892)	<.001	1.07 (1.09)	0.837 (0.769-0.911)	<.001
Antipsychotics	0.17 (0.44)	0.891 (0.828-0.959)	.002	0.60 (0.71)	0.896 (0.845-0.950)	<.001
Anxiolytics	0.05 (0.20)	0.756 (0.672-0.850)	<.001	0.08 (0.23)	0.791 (0.640-0.978)	.03
Antidepressants	0.12 (0.31)	0.753 (0.683-0.830)	<.001	0.28 (0.44)	0.696 (0.332-1.457)	.34
Psychostimulants	0.05 (0.20)	0.849 (0.721-1.001)	.05	0.08 (0.24)	0.721 (0.237-2.194)	.57

Abbreviations: ATC, Anatomic Therapeutic Chemical; IRR, incidence rate ratio; OR, odds ratio.

^a^ Analysis included Medicaid Analytic eXtract claims data (January 1, 2007, to December 31, 2012). Regressions were adjusted for covariates listed in the Methods section. Difference-in-differences estimates were calculated among all individuals with disabilities in drug cap states (n = 8214) compared with individuals living in comparison states (n = 19 832) and the subgroup of individuals with a serious mental illness in drug cap states (n = 1178) and comparison states (n = 2957). Pre–age 21 years means were calculated in the drug cap states (Arkansas and Texas) by first averaging monthly measures in the 12 months before age 21 years for each individual and then averaging across all individuals. Prescription drug outcomes were measured in each of the 12 calendar months before and after the individual became age 21 years, and prescriptions in the month of the 21st birthday were not included. All results are from the coefficient on the interaction between treated indicator variable and post-policy indicator. All results for count outcomes (total prescriptions and spending) are reported as IRRs from the 0-inflated negative binomial models; results for binary outcomes (>3 prescriptions) are reported as ORs from the logistic models.

A sharp decrease in the mean number of prescriptions to treat a mental health condition at age 21 years was also observed in drug cap states (Figure, C). Relative to comparison states, being older than 21 years in a drug cap state was associated with 16.5% (95% CI, −21.9% to −10.8%; *P* < .001) lower total monthly prescriptions for drugs to treat a mental health condition as well as lower total monthly prescriptions for antipsychotics, anxiolytics, and antidepressants (Table 2). The age cutoff was not associated with total spending on prescription drugs but was associated with 13.3% (95% CI, 3.8% to 23.7%; *P* = .005) higher mean spending per prescription (Table 2).

Individuals with a serious mental illness filled multiple prescriptions per month before becoming age 21 years in both the drug cap (2.63 [2.65]) and comparison (3.39 [3.23]) states, but a substantial decrease in these prescriptions at age 21 years was evident in drug cap states only (Figure, B; eTable 4 in the Supplement). Relative to comparison states, being older than 21 years in a drug cap state was associated with 19.7% (95% CI, −22.9% to −16.3%; *P* < .001) fewer monthly prescriptions and 46.5% (95% CI, −51.2% to −41.4%; *P* < .001) fewer months with more than 3 prescriptions (Table 2).

Monthly prescriptions for drugs used to treat mental health conditions also visibly decreased at age 21 years among individuals with a serious mental illness in drug cap states (Figure, D). Relative to comparison states, being older than 21 years in a drug cap state was associated with 16.3% (95% CI −23.1% to −8.9%; *P* < .001) lower monthly prescriptions for drugs to treat a mental health condition as well as fewer prescriptions for antipsychotics and antidepressants (Table 2). Being older than 21 years was also associated with 5.8% (95%, CI, −10.1% to −1.3%; *P* = .01) lower total monthly spending on prescription drugs and 9.8% (95% CI, 4.0% to 16.0%, *P* < .001) higher spending per prescription (Table 2).

### Health Care Use

For all young adults with disabilities relative to comparison states, being older than 21 years in a drug cap state was associated with a 13.6% (95% CI, 1.9%-26.6%; *P* = .02) higher proportion of patients with any quarterly inpatient admission but not with total inpatient length of stay or total inpatient spending (Table 3). Being age 21 years in a drug cap state was not associated with any significant changes in total emergency department visits, spending on emergency department visits, or total combined spending on prescription drugs, inpatient visits, and emergency department visits.

**Table 3.** Changes in Quarterly Use of Health Care Services After Exposure to the Drug Cap Policy at Age 21 Years^a^

Health care use outcomes	Difference-in-differences estimates					
	All individuals with disabilities (N = 28 046)			Individuals with disabilities and a serious mental illness (n = 4135)		
	Pre–age 21 y in drug cap states, mean (SD)	IRR/OR (95% CI)	*P* value	Pre–age 21 y in drug cap states, mean (SD)	IRR/OR (95% CI)	*P* value
Quarterly ED visits						
Total ED visits	0.43 (1.38)	0.959 (0.888-1.035)	.28	0.55 (1.80)	0.951 (0.766-1.182)	.65
Total ED spending, $	131.00 (640.12)	0.969 (0.916-1.025)	.27	152.42 (690.98)	1.026 (0.906-1.163)	.68
Quarterly IP visits						
Any IP admission, %	2.43 (9.85)	1.136 (1.019-1.266)	.02	4.39 (12.42)	1.177 (0.930-1.488)	.17
Total IP length of stay	0.21 (1.50)	0.917 (0.784-1.072)	.28	0.36 (1.22)	0.964 (0.757-1.226)	.76
Total IP spending, $	546.42 (5198.68)	1.168 (0.986-1.384)	.07	515.99 (1499.16)	1.106 (0.876-1.397)	.34
Quarterly spending						
Total ED, IP, and prescription spending, $	1595.16 (6349.58)	0.964 (0.863-1.076)	.51	2364.36 (3266.02)	1.014 (0.934-1.101)	.74

Abbreviations: ED, emergency department; IP, inpatient; IRR, incidence rate ratio; OR, odds ratio.

^a^ Analysis included Medicaid Analytic eXtract claims data (January 1, 2007, to December 31, 2012). Regressions were adjusted for covariates listed in the Methods section. Difference-in-differences estimates were calculated among all individuals with disabilities in drug cap states (n = 8214) compared with individuals living in comparison states (n = 19 832) and the subgroup of individuals with a serious mental illness in drug cap states (n = 1178) and comparison states (n = 2957). Pre–age 21 years means were calculated in the drug cap states (Arkansas and Texas) by first averaging monthly measures in the 12 months before age 21 years for each individual and then averaging across all individuals. Prescription drug outcomes were measured among all individuals on a monthly basis; health care resource use was measured on a quarterly basis before and after the individual became age 21 years. Total spending includes all spending on prescription drugs as well as inpatient and emergency department visits. All results are from the coefficient on the interaction between treated indicator variable and post-policy indicator. All results for count outcomes (total visits and spending) are reported as IRRs from the 0-inflated negative binomial models; results for binary outcomes (any emergency or any inpatient visit) are reported as ORs from the logistic models.

Among all individuals with a serious mental illness, the drug cap was not associated with any changes in quarterly inpatient admission or emergency department visits or spending. In addition, the drug cap was not associated with any significant changes in the total combined spending on prescription drugs, inpatient visits, and emergency department visits.

### Sensitivity Analyses

In our sensitivity analyses, we found that the drug cap policy was associated with a lower total days’ supply of medications across all prescriptions but higher mean days’ supply per prescription. In addition, the drug cap policy was associated with fewer prescriptions in all quartiles of spending; however, the percent change in monthly prescriptions was largest in the lowest spending quartile (eTable 5 in the Supplement).

Results for the main analyses were similar when adjusted for clustering by state (eTable 6 in the Supplement). There were no significant differences in linear trends for all of the outcomes of interest before exposure to the drug cap at age 21 (eTable 7 in the Supplement).

## Discussion

This study noted that exposure to a 3-drug prescription limit at age 21 years was associated with lower monthly prescriptions among young Medicaid beneficiaries with disabilities as well as those with a serious mental illness. The drug cap policies restricted prescriptions for many individuals, with decreases in important medications used to treat mental illness, including antipsychotics, anxiolytics, and antidepressants. Despite these decreases in prescriptions, the drug cap policies were not associated with lower spending on prescriptions among all individuals with disabilities and only $33 lower monthly prescription drug spending among those with a serious mental illness. In addition, the results suggest that the drug cap policy may be associated with higher rates of inpatient care.

These decreases in prescriptions may have substantial implications for the treatment of both physical and mental health conditions. Earlier research has suggested the importance of adherence to prescription drugs for depression and schizophrenia and also for reducing overall medical costs and use of health care services.^25^ Reducing the burden of symptoms is particularly important in this population of patients with disabilities owing to the high prevalence of comorbid physical and mental health conditions. In addition to increasing the costs associated with mental health conditions, nonadherence to drugs, such as antidepressants and antipsychotics, can complicate the treatment of other chronic conditions. For example, patients with diabetes who have symptoms of depression are less likely to achieve glycemic control compared with patients without symptoms of depression.^26^

By reducing access to necessary prescription drugs, the drug cap policies may also be associated with decreases in patient quality of life. Mental health conditions are among the leading causes of disability-adjusted life years, particularly among young adults, and exposure to the drug cap policies at age 21 is likely to limit access to treatment and exacerbate the detrimental effects of these conditions.^27,28^ For example, symptoms of mental health conditions, including depression and bipolar disorder, are associated with decreases in productivity and functional status.^26,29^ Furthermore, nonadherence among patients with psychiatric disorders has been linked to increases in the risk of incarceration, suicide, and premature mortality.^30,31^ Additional research is necessary to understand potential long-term consequences of the drug cap policies on quality of life among young adults.

Combined with the decreases in necessary medications, the lack of savings from the drug cap policy suggests that, in our sample of Medicaid beneficiaries with disabilities, the policy failed to provide measurable benefits for state Medicaid programs. The absence of a significant decrease in prescription drug spending among all patients can be explained in part by higher average spending per prescription and larger percent decreases for prescriptions in the lowest spending quartile. These findings suggest that beneficiaries may be either discontinuing less expensive lower-value drugs or paying for less expensive prescriptions out of pocket. In addition, the increase in days’ supply per prescription suggests that health care professionals may be prescribing a greater quantity of medication to reduce the influence of the drug cap, which may also increase spending per prescription. This finding that the prescription drug cap was not associated with any savings but was associated with increased health care use is consistent with previous literature reporting that other drug rationing policies, including prior authorization and step therapy, generally do not save state Medicaid programs money but instead contribute to worse patient outcomes.^32^ Together, these results suggest that states should reevaluate the use of drug-rationing policies owing to the potential for adverse effects among individuals with disabilities.

### Limitations

This study had several limitations. First, the MAX data only include claims for Medicaid-paid prescriptions, so out-of-pocket prescription purchases are not documented. However, prior research on Medicaid copayment policies suggests that, owing to the high out-of-pocket costs of prescription drugs, some beneficiaries may not pay for prescriptions above the 3-drug limit.^33^ Second, although we saw an increase in days’ supply, we could not analyze changes in dosage to determine whether patients may be splitting pills to reduce the number of prescriptions per month. Third, the MAX data were only available through 2012 at the time this study was conducted. Although the number of beneficiaries with disabilities in Texas subject to the drug cap has decreased due to the shift to comprehensive Medicaid Managed Care, these findings are still relevant because the 3-drug limits remain in place for all individuals with disabilities in Arkansas. Fourth, we limited our sample to fee-for-service Medicaid. In some states, beneficiaries in comprehensive Medicaid Managed Care are subject to drug caps and results may differ among these individuals if insurers have the incentive to prevent spillover effects, including costly hospitalizations. Fifth, we were unable to observe potential long-term outcomes that would be affected by reduced access to important medications, including health outcomes and work productivity.

Sixth, we did not include total outpatient visits because the place of service and type of service codes varied both across states and over time, which would affect the validity of the analyses. However, we expect that the drug cap would have the largest spillover effects on emergency department and inpatient visits, which were measured consistently in the MAX data.^34,35^

Seventh, results may be confounded by benefits changes occurring at age 21 years. For example, in most state Medicaid programs, individuals lose or have reduced dental, hearing, vision, and chiropractic benefits at age 21 years. Reduced dental benefits could lower the number of prescriptions for opioids and antibiotics, and reduced chiropractic benefits may increase prescriptions for opioids.^36,37^ Because of these changes in benefits, we did not analyze variations in total spending on all services. In addition, other policy changes at age 21 years, such as the ability to purchase alcohol and firearms, may increase the use of inpatient and emergency department care. However, these policies are not expected to affect the use of prescription drugs.

## Conclusions

In this cohort study of young US adults with disabilities, drug cap policies were associated with lower monthly prescriptions overall and for drugs used to treat mental health conditions and higher rates of hospitalization among individuals with disabilities. This lower number of prescriptions combined with higher rates of inpatient admissions suggest that the drug cap policies used by many states may be limiting access to necessary health care services and increasing the risk of hospitalization without any significant changes in prescription drug spending.

## Notes

Supplement. **eMethods.** Statistical Analysis **eTable 1.** Anatomic Therapeutic Chemical Classification for Drugs Used to Treat Mental Health Conditions **eTable 2.** Sample Selection **eTable 3.** Use of Prescription Drugs and Health Care Services Before and After Implementation of Drug Cap Policy at Age 21 Among All Individuals With Disabilities **eTable 4.** Use of Prescription Drugs and Health Care Services Before and After Implementation of Drug Cap Policy at Age 21 Among Individuals With Disabilities and a Serious Mental Illness **eTable 5.** Changes in Monthly Prescriptions After the Implementation of the Drug Cap Policy at Age 21 **eTable 6.** Changes in Prescriptions and Health Care Use With Standard Errors Clustered by State **eTable 7.** Pre-Policy Period Test for Parallel Trends
