# Supplementary material for: Association Between Medicaid Prescription Drug Limits and Access to Medications and Health Care Use Among Young Adults With Disabilities
Source: JAMA Health Forum. 2021 Jun 17;2(6):e211048. doi: 10.1001/jamahealthforum.2021.1048 (PMC8796920; doi:10.1001/jamahealthforum.2021.1048)
Supplement: Supplement. — eMethods. Statistical Analysis eTable 1. Anatomic Therapeutic Chemical Classification for Drugs Used to Treat Mental Health Conditions eTable 2. Sample Selection eTable 3. Use of Prescription Drugs and Health Care Services Before and After Implementation of Drug Cap Policy at Age 21 Among All Individuals With Disabilities eTable 4. Use of Prescription Drugs and Health Care Services Before and After Implementation of Drug Cap Policy at Age 21 Among Individuals With Disabilities and a Serious Mental Illness eTable 5. Changes in Monthly Prescriptions After the Implementation of the Drug Cap Policy at Age 21 eTable 6. Changes in Prescriptions and Health Care Use With Standard Errors Clustered by State eTable 7. Pre-Policy Period Test for Parallel Trends [file jamahealthforum-e211048-s001.pdf]

Supplementary Online Content

Geiger CK, Cohen JL, Sommers BD. Association between Medicaid prescription drug limits and access to medications and health care use among young adults with disabilities. *JAMA Health Forum*. 2021;2(6):e211048. doi:10.1001/jamahealthforum.2021.1048

eMethods. Statistical Analysis

eTable 1. Anatomic Therapeutic Chemical Classification for Drugs Used to Treat Mental Health Conditions

eTable 2. Sample Selection

eTable 3. Use of Prescription Drugs and Health Care Services Before and After Implementation of Drug Cap Policy at Age 21 Among All Individuals with Disabilities

eTable 4. Use of Prescription Drugs and Health Care Services Before and After Implementation of Drug Cap Policy at Age 21 Among Individuals with Disabilities and a Serious Mental Illness

eTable 5. Changes in Monthly Prescriptions After the Implementation of the Drug Cap Policy at Age 21

eTable 6. Changes in Prescriptions and Health Care Use with Standard Errors Clustered by State

eTable 7. Pre-Policy Period Test for Parallel Trends

This supplementary material has been provided by the authors to give readers additional information about their work.

© 2021 Geiger CK et al. *JAMA Health Forum.*

eMethods. Statistical Analysis

Main Analyses

The regression model for the difference-in-differences analyses was specified as:

$$y_{ims} = \alpha_o + \beta_1 Age21_{im} + \beta_2 DrugCap + \beta_3 DrugCap_s * Age21_{im} + +X_i + \theta_s + \gamma_t + \epsilon_{ims}$$

In the regression model, the variable $Age21_{im}$ is a dummy variable that indicates whether the individual was age 21 or older in that month or quarter and $DrugCap_s$ is an indicator for whether the individual resides in one of the drug cap states (Arkansas or Texas). The coefficient of interest is β_3 which identifies the change in the outcome of interest before versus after age 21 in drug cap states compared with comparison states. All outcomes were calculated in each month or quarter in the 12 calendar months before and after the month of an individual's 21st birthday. The month of an individual's twenty-first birthday was defined as a transition period and not included in the analyses. The regression adjusted for individual sex and race/ethnicity (white, black, or other) as well as whether the individual lived in an urban county (Rural Urban Continuum Codes 1-3) prior to turning age 21. Heteroskedastic-robust standard errors were clustered at the individual level. The functional form of the model differed based on the distribution of the outcomes of interest. Regressions were estimated using a zero-inflated negative binomial model for skewed count outcomes with a high proportion of zeros (i.e., prescriptions, total emergency department visits, inpatient length of stay, and spending) while logistic regressions were used for binary outcomes (i.e., any prescription, more than 3 prescriptions, and any inpatient visit).

All analyses were implemented using STATA/MP version 15. Results with p-values <0.05 were considered statistically significant.

© 2021 Geiger CK et al. *JAMA Health Forum.*

Test for Parallel Trends

The difference-in-difference analysis relies on the assumption that the trends in the outcomes of interest would have been parallel in the absence of the policy change at age 21. Although this "parallel trends" assumption cannot be tested directly, we tested whether this assumption is plausible by testing whether the trends in the outcomes were parallel in the pre-policy period.

The regression model for the parallel trends test was specified as:

$$y_{ims} = \alpha_o + \beta_1 AMonths21_{im} + \beta_2 DrugCap_s + \beta_3 DrugCap_s * Months21_{im} + +X_i + \theta_s + \gamma_t + \epsilon_{ims}$$

In the regression model, the variable $Months21_{im}$is a continuous measure of the number of months until the individual turns age 21 (i.e., values range from 1-12) and $DrugCap_s$ is an indicator for whether the individual resides in one of the drug cap states (Arkansas or Texas). β_3 represents any difference in the trends in the outcomes in the drug cap states compared with the comparison states prior to age 21. The regression only used data for the 12 calendar months before the individual turned age 21. Similar to the main analyses, the model also included state and calendar month and year fixed-effects as well as individual characteristics and heteroskedastic-robust standard errors that were adjusted for clustering at the individual level. Results for the parallel trends analyses are included below in eTable 4.

© 2021 Geiger CK et al. *JAMA Health Forum.*

eTable 1: Anatomic Therapeutic Chemical Classification for Drugs Used to Treat Mental Health Conditions

Type of Prescription Drug	ATC Class
Drugs to treat mental health conditions	N05, N06
Psycholeptics	N05
Antipsychotics	N05A
Anxiolytics	N05B
Hypnotics and sedatives	N05C
Psychoanaleptics	N06
Antidepressants	N06A
Psychostimulants	N06B
Psycholeptics and psychoanaleptics	N06C
Abbreviations: ATC = Anatomic Therapeutic Chemical.	

© 2021 Geiger CK et al. *JAMA Health Forum.*

eTable 2: Sample Selection

Sample Selection Step	Drug Cap States	Comparison States
1. All individuals who were enrolled in full-benefit, fee-for-service Medicaid and turned age 21 in 2009-2011[1]	353,006	734,742
2. 12 months of continuous eligibility before and after turning age 21	12,734	37,193
3. Not eligible for Medicaid due to foster care	12,661	36,049
4. Not pregnant in 14 months prior to age 21[2]	11,920	34,618
5. Not in long term care in 12 months prior to age 21[3]	11,263	33,715
6. Eligible for Medicaid due to a disability prior to age 21	10,329	25,547
7. Not dually enrolled in Medicare in 12 months before or after turning age 21	8,214	19,832
**Notes:**		
1. Eligibility for Medicaid and enrollment in Medicare were identified in the Personal Summary file in the MAX data.		
2. Individuals who were pregnant were identified using diagnosis and procedure codes for pregnancy or delivery. Individuals were excluded if they were pregnant or delivered in the 14 months before age 21 instead of 12 months since individuals are not subject to the drug cap during pregnancy as well as during the 60-day postpartum period.		
3. Individuals in long term care were identified using any services in the Long Term Care file in the MAX data.		

© 2021 Geiger CK et al. *JAMA Health Forum.*

eTable 3: Use of Prescription Drugs and Health Care Services Before and After Implementation of Drug Cap Policy at Age 21 Among All Individuals with Disabilities

	**All Individuals with Disabilities**			
	**Drug Cap States**		**Comparison States**	
	**Pre**	**Post**	**Pre**	**Post**
**N**	8,214	8,214	19,832	19,832
**Average Monthly Prescription Drug Use**				
Any prescription, n (%)	6,483 (78.9%)	6,446 (78.5%)	14,968 (75.5%)	15,059 (75.9%)
Total prescriptions, mean (SD)	1.58 (2.16)	1.39 (1.91)	1.83 (2.61)	1.90 (2.66)
Total days' supply across all prescriptions, mean (SD)	43.22 (62.57)	40.24 (57.56)	47.61 (68.03)	50.21 (70.86)
Total days' supply per prescription, mean (SD)	24.54 (12.93)	26.30 (14.41)	24.18 (10.25)	24.62 (10.73)
% with > 3 prescriptions in month, mean (SD)	16.54% (28.59)	10.81% (25.83)	19.05% (31.71)	19.95% (32.47)
Any month with > 3 prescriptions, n (%)	3,436 (41.8%)	1,906 (23.2%)	8,272 (41.7%)	8,463 (42.7%)
**Any Prescription in ATC Class in Month, n (%)**				
Drugs for mental health conditions (N05/N06)	3,249 (39.6%)	3,156 (38.4%)	8,151 (41.1%)	8,227 (41.5%)
Psycholeptics (N05)	2,472 (30.1%)	2,386 (29.0%)	6,270 (31.6%)	6,321 (31.9%)
Antipsychotics (N05A)	1,720 (20.9%)	1,699 (20.7%)	4,540 (22.9%)	4,500 (22.7%)
Anxiolytics (N05B)	1,047 (12.7%)	973 (11.8%)	2,664 (13.4%)	2,806 (14.1%)
Hypnotics and sedatives (N05C)	400 (4.9%)	370 (4.5%)	936 (4.7%)	996 (5.0%)
Psychoanaleptics (N06)	2,098 (25.5%)	2,049 (24.9%)	5,360 (27.0%)	5,494 (27.7%)
Antidepressants (N06A)	1,683 (20.5%)	1,671 (20.3%)	4,313 (21.7%)	4,552 (23.0%)
Psychostimulants (N06B)	720 (8.8%)	622 (7.6%)	1,887 (9.5%)	1,773 (8.9%)
**Average Monthly Prescriptions per ATC Class, mean (SD)**				
Drugs for mental health conditions (N05/N06)	0.40 (0.78)	0.37 (0.73)	0.54 (1.06)	0.56 (1.06)
Psycholeptics (N05)	0.23 (0.54)	0.22 (0.52)	0.32 (0.73)	0.32 (0.73)
Antipsychotics (N05A)	0.17 (0.44)	0.16 (0.43)	0.24 (0.63)	0.24 (0.62)
Anxiolytics (N05B)	0.05 (0.20)	0.05 (0.19)	0.06 (0.22)	0.06 (0.23)
Hypnotics and sedatives (N05C)	0.01 (0.09)	0.01 (0.09)	0.02 (0.12)	0.02 (0.13)
Psychoanaleptics (N06)	0.17 (0.39)	0.15 (0.35)	0.23 (0.52)	0.23 (0.52)
Antidepressants (N06A)	0.12 (0.31)	0.11 (0.28)	0.16 (0.42)	0.17 (0.42)
Psychostimulants (N06B)	0.05 (0.20)	0.04 (0.18)	0.07 (0.25)	0.06 (0.25)
**Average Monthly Health Care Services, mean (SD)**				
Total IP admittances	0.01 (0.05)	0.01 (0.05)	0.01 (0.07)	0.01 (0.08)
Total IP length of stay	0.07 (0.50)	0.08 (0.39)	0.11 (0.81)	0.11 (0.84)
Total ED visits	0.14 (0.46)	0.16 (0.57)	0.06 (0.14)	0.06 (0.15)
**Average Monthly Health Care Spending (USD), mean (SD)**				
Prescription drugs	305.92 (1,019.98)	291.85 (1,465.99)	349.43 (4,118.24)	338.25 (2,760.08)
IP services	182.14 (1,732.89)	157.77 (873.64)	186.58 (1,439.06)	187.34 (1,484.69)

© 2021 Geiger CK et al. *JAMA Health Forum.*

ED visits	43.67 (213.37)	49.20 (241.80)	12.55 (49.78)	13.52 (70.14)
Abbreviations: SD = standard deviation; ATC = Anatomic Therapeutic Chemical; IP = inpatient; ED = emergency department; USD = 2020 United States dollars.				
**Notes:** The sample of all individuals with disabilities includes all Medicaid beneficiaries who were eligible for Medicaid due to a disability prior to turning age 21 and were continuously enrolled in fee-for-service Medicaid in the year before and after turning age 21. The treatment group includes all individuals residing in Arkansas and Texas who were eligible for the drug cap policy at age 21. The control group includes all individuals residing in Colorado, Connecticut, Idaho, Missouri, Nebraska, New Hampshire, Nevada, Virginia, Washington, and Wisconsin who were not eligible for a drug cap policy at age 21. The pre-period includes all prescription drug and health care services in the 12 calendar months before an individual turns age 21 and post-period includes all prescription drug and health care services in the 12 calendar months after an individual turns age 21. Means were calculated by first averaging monthly measures in the 12 months before and after age 21 for each individual and then averaging across all individuals.				

© 2021 Geiger CK et al. *JAMA Health Forum.*

eTable 4: Use of Prescription Drugs and Health Care Services Before and After Implementation of Drug Cap Policy at Age 21 Among Individuals with Disabilities and a Serious Mental Illness

	Individuals with Disabilities and a Serious Mental Illness			
	Drug Cap States		Comparison States	
	Pre	Post	Pre	Post
N	1,178	1,178	2,957	2,957
**Average Monthly Prescription Drug Use**				
Any prescription, n (%)	1,108 (94.1%)	1,090 (92.5%)	2,797 (94.6%)	2,768 (93.6%)
Total prescriptions, mean (SD)	2.63 (2.65)	2.26 (2.50)	3.39 (3.23)	3.46 (3.40)
Total days' supply across all prescriptions, mean (SD)	76.35 (79.16)	68.93 (75.53)	88.10 (80.91)	91.13 (87.02)
Total days' supply per prescription, mean (SD)	28.52 (12.34)	30.08 (14.05)	25.74 (6.98)	25.76 (7.46)
% with > 3 prescriptions in month, mean (SD)	29.95% (34.87)	19.05% (33.77)	38.71% (37.81)	39.08% (38.45)
Any month with > 3 prescriptions, n (%)	776 (65.9%)	404 (34.3%)	2,154 (72.8%)	2,098 (71.0%)
**Any Prescription in ATC Class in Month, n (%)**				
Drugs for mental health conditions (N05/N06)	976 (82.9%)	928 (78.8%)	2,531 (85.6%)	2,444 (82.7%)
Psycholeptics (N05)	904 (76.7%)	841 (71.4%)	2,320 (78.5%)	2,225 (75.2%)
Antipsychotics (N05A)	835 (70.9%)	771 (65.4%)	2,180 (73.7%)	2,070 (70.0%)
Anxiolytics (N05B)	255 (21.6%)	239 (20.3%)	743 (25.1%)	767 (25.9%)
Hypnotics and sedatives (N05C)	131 (11.1%)	114 (9.7%)	320 (10.8%)	329 (11.1%)
Psychoanaleptics (N06)	628 (53.3%)	586 (49.7%)	1,718 (58.1%)	1,696 (57.4%)
Antidepressants (N06A)	550 (46.7%)	518 (44.0%)	1,481 (50.1%)	1,499 (50.7%)
Psychostimulants (N06B)	179 (15.2%)	139 (11.8%)	557 (18.8%)	477 (16.1%)
**Average Monthly Prescriptions per ATC Class, mean (SD)**				
Drugs for mental health conditions (N05/N06)	1.07 (1.09)	0.96 (1.04)	1.59 (1.62)	1.55 (1.57)
Psycholeptics (N05)	0.72 (0.81)	0.66 (0.80)	1.05 (1.19)	1.02 (1.18)
Antipsychotics (N05A)	0.60 (0.71)	0.55 (0.70)	0.89 (1.08)	0.86 (1.05)
Anxiolytics (N05B)	0.08 (0.23)	0.08 (0.22)	0.12 (0.30)	0.12 (0.30)
Hypnotics and sedatives (N05C)	0.03 (0.13)	0.03 (0.14)	0.05 (0.18)	0.05 (0.18)
Psychoanaleptics (N06)	0.36 (0.52)	0.30 (0.46)	0.53 (0.76)	0.52 (0.71)
Antidepressants (N06A)	0.28 (0.44)	0.23 (0.39)	0.40 (0.65)	0.41 (0.61)
Psychostimulants (N06B)	0.08 (0.24)	0.07 (0.22)	0.13 (0.33)	0.11 (0.32)
**Average Monthly Health Care Services, mean (SD)**				
Total IP admittances	0.02 (0.05)	0.02 (0.06)	0.03 (0.09)	0.03 (0.11)
Total IP length of stay	0.12 (0.41)	0.14 (0.50)	0.22 (0.93)	0.22 (1.18)
Total ED visits	0.18 (0.60)	0.20 (0.82)	0.12 (0.21)	0.12 (0.22)

© 2021 Geiger CK et al. *JAMA Health Forum.*

**Average Monthly Health Care Spending (USD), mean (SD)**				
Prescription drugs	565.32 (777.01)	527.99 (755.44)	586.22 (902.10)	578.49 (908.61)
IP services	172.00 (499.72)	190.74 (782.22)	263.19 (1,126.98)	248.39 (1,215.90)
ED visits	50.81 (230.33)	62.72 (301.28)	31.23 (86.05)	30.43 (84.23)
Abbreviations: SD = standard deviation; ATC = Anatomic Therapeutic Chemical; IP = inpatient; ED = emergency department; USD = 2020 United States dollars.				
**Notes:** The sample of all individuals with disabilities includes all Medicaid beneficiaries who were eligible for Medicaid due to a disability prior to turning age 21 and were continuously enrolled in fee-for-service Medicaid in the year before and after turning age 21. The serious mental illness subgroup includes all individuals with disabilities who were diagnosed with schizophrenia and psychotic disorders or bipolar disorder at any time prior to turning age 21. The treatment group includes all individuals residing in Arkansas and Texas who were eligible for the drug cap policy at age 21. The control group includes all individuals residing in Colorado, Connecticut, Idaho, Missouri, Nebraska, New Hampshire, Nevada, Virginia, Washington, and Wisconsin who were not eligible for a drug cap policy at age 21. The pre-period includes all prescription drug and health care services in the 12 calendar months before an individual turns age 21 and post-period includes all prescription drug and health care services in the 12 calendar months after an individual turns age 21.				

© 2021 Geiger CK et al. *JAMA Health Forum.*

eTable 5: Changes in Monthly Prescriptions After the Implementation of the Drug Cap Policy at Age 21

	Difference-in-Differences Estimates								
	All Individuals with Disabilities (N=28,046)					Individuals with Disabilities and a Serious Mental Illness (N=4,135)			
**Prescriptions**	**Pre-age 21 mean (SD) in drug cap states**	**IRR**	**95% CI**	**P-value**		**Pre-age 21 mean (SD) in drug cap states**	**IRR**	**95% CI**	**P-value**
**Prescription days' supply**									
Total days' supply for all prescriptions	43.22 (62.57)	0.892	(0.879, 0.905)	<0.001		76.35 (79.16)	0.868	(0.842, 0.895)	<0.001
Average days' supply per prescription	24.54 (12.93)	1.047	(1.038, 1.056)	<0.001		28.52 (12.34)	1.045	(1.026, 1.065)	<0.001
**Monthly prescriptions by spending quartile**									
$0-9	0.12 (0.31)	0.688	(0.610, 0.775)	<0.001		0.19 (0.42)	0.655	(0.531, 0.808)	<0.001
$10-29	0.55 (0.86)	0.764	(0.733, 0.797)	<0.001		0.91 (1.11)	0.736	(0.676, 0.802)	<0.001
$30-159	0.47 (0.79)	0.829	(0.793, 0.868)	<0.001		0.65 (0.84)	0.858	(0.746, 0.987)	0.03
≥ $160	0.44 (0.81)	0.898	(0.860, 0.937)	<0.001		0.88 (1.08)	0.917	(0.853, 0.986)	0.02
**Monthly prescriptions by branded status**									
Branded	0.61 (0.99)	0.850	(0.819, 0.882)	<0.001		1.04 (1.23)	0.850	(0.788, 0.916)	<0.001
Generic	0.97 (1.42)	0.766	(0.744, 0.789)	<0.001		1.59 (1.75)	1.009	(0.969, 1.051)	0.66

Abbreviations: SD = standard deviation; IRR =incidence rate ratio; CI = confidence interval; ATC = Anatomic Therapeutic Chemical; IP = inpatient; ED = emergency department; USD = 2020 United States dollars.

Source: Author's analysis of Medicaid Analytic eXtract (MAX) claims data (2007-2012).

Notes: Regressions adjusted for covariates listed in methods. Difference-in-differences estimates were calculated among all individuals with disabilities in drug cap states (n=8,214) compared with individuals living in comparison states (n=19,832) and the subgroup of individuals with a serious mental illness in drug cap states (n=1,178) and comparison states (n=2,957). Pre-age 21 means were calculated in the drug cap states (Arkansas and Texas) among all individuals in the 12 calendar months prior to turning age 21. Pre-age 21 means were calculated by first averaging monthly measures in the 12 months prior to age 21 for each individual and then averaging across all individuals. Prescription drug outcomes were measured among all individuals on a monthly basis while health care resource use was measured on a quarterly basis before and after the individual turned age 21. Total spending includes all spending on prescription drugs as well as inpatient and emergency department visits. All results are from the coefficient on the interaction between treated indicator variable and post-policy indicator. All results for count outcomes (total prescriptions) are reported as incidence rate ratios (IRR) from the zero-inflated negative binomial models.

© 2021 Geiger CK et al. *JAMA Health Forum.*

eTable 6: Changes in Prescriptions and Health Care Use with Standard Errors Clustered by State

	Difference-in-Differences Estimates						
	All Individuals with Disabilities (N=28,046)				Individuals with Disabilities and a Serious Mental Illness (N=4,135)		
	OR/IRR	95% CI	P-value		OR/IRR	95% CI	P-value
**Monthly prescriptions overall**							
Total prescriptions	0.804	(0.772, 0.838)	<0.001		0.803	(0.771, 0.837)	<0.001
> 3 prescriptions in month	0.576	(0.515, 0.646)	<0.001		0.535	(0.488, 0.586)	<0.001
**Monthly prescriptions by ATC class**							
All mental health drugs (N05/N06)	0.835	(0.792, 0.879)	<0.001		0.837	(0.769, 0.911)	<0.001
Antipsychotics (N05A)	0.891	(0.849, 0.936)	<0.001		0.896	(0.845, 0.950)	<0.001
Anxiolytics (N05B)	0.756	(0.668, 0.855)	<0.001		0.791	(0.640, 0.978)	0.03
Antidepressants (N06A)	0.753	(0.697, 0.814)	<0.001		0.696	(0.332, 1.457)	0.34
Psychostimulants (N06B)	0.849	(0.781, 0.924)	<0.001		0.721	(0.237, 2.194)	0.57
**Monthly prescription drug spending**							
Total prescription spending	0.996	(0.943, 1.053)	0.89		0.942	(0.899, 0.987)	0.01
Total spending per prescription	1.133	(1.092, 1.176)	<0.001		1.098	(1.040, 1.160)	<0.001
**Quarterly health care resource use**							
Total ED visits	0.959	(0.888, 1.035)	0.28		0.951	(0.766, 1.182)	0.65
Total ED spending (USD)	0.969	(0.916, 1.025)	0.27		1.026	(0.906, 1.163)	0.68
Any IP visit	1.136	(1.019, 1.266)	0.02		1.177	(0.930, 1.488)	0.17
Total IP length of stay	0.917	(0.784, 1.072)	0.28		0.964	(0.757, 1.226)	0.76
Total IP spending (USD)	1.168	(0.986, 1.384)	0.07		1.106	(0.876, 1.397)	0.40
Total (ED, IP, prescription) spending (USD)	0.964	(0.863, 1.076)	0.51		1.014	(0.934, 1.101)	0.74

Abbreviations: OR=odds ratio; IRR =incidence rate ratio; CI = confidence interval; ATC = Anatomic Therapeutic Chemical; IP = inpatient; ED = emergency department; USD = 2020 United States dollars.

Source: Author's analysis of Medicaid Analytic eXtract (MAX) claim data (2007-2012).

Notes: Regressions adjusted for covariates listed in methods. Difference-in-differences estimates were calculated among all individuals with disabilities in drug cap states (n=8,214) compared with individuals living in comparison states (n=19,832) and the subgroup of individuals with a serious mental illness in drug cap states (n=1,178) and comparison states (n=2,957). Pre-age 21 means were calculated in the drug cap states (Arkansas and Texas) among all individuals in the 12 calendar months prior to turning age 21. Pre-age 21 means were calculated by first averaging monthly measures in the 12 months prior to age 21 for each individual and then averaging across all individuals. Prescription drug outcomes were measured in each of the 12 calendar months before and after the individual turned age 21 and prescriptions in the month of the twenty-first birthday were not included. All results are from the coefficient on the interaction between treated indicator variable and post-policy indicator. All results for count outcomes (total prescriptions and spending) are reported as incidence rate ratios (IRR) from the zero-inflated negative binomial models while results for binary outcomes (more than 3 prescriptions) are reported as odds ratios (OR) from the logistic models.

© 2021 Geiger CK et al. *JAMA Health Forum.*

eTable 7: Pre-Policy Period Test for Parallel Trends

	Pre-Policy Trends						
	**All Individuals with Disabilities (N=28,046)**				**Individuals with Disabilities and a Serious Mental Illness (N=4,135)**		
	**OR/IRR**	**95% CI**	**P-value**		**OR/IRR**	**95% CI**	**P-value**
**Monthly prescriptions overall**							
Total prescriptions	0.998	(0.995, 1.000)	0.08		0.997	(0.992, 1.002)	0.26
> 3 prescriptions in month	0.998	(0.993, 1.002)	0.30		0.991	(0.980, 1.001)	0.08
**Monthly prescriptions by ATC class**							
All mental health drugs (N05/N06)	1.001	(0.996, 1.006)	0.81		0.998	(0.991, 1.005)	0.53
Antipsychotics (N05A)	1.003	(0.994, 1.013)	0.52		0.997	(0.990, 1.005)	0.50
Anxiolytics (N05B)	0.987	(0.950, 1.027)	0.52		1.006	(0.970, 1.042)	0.75
Antidepressants (N06A)	1.004	(0.994, 1.015)	0.42		0.988	(0.966, 1.012)	0.32
Psychostimulants (N06B)	1.002	(0.977, 1.028)	0.89		0.992	(0.941, 1.046)	0.77
**Monthly prescription drug spending**							
Total prescription spending	0.998	(0.990, 1.005)	0.52		1.000	(0.994, 1.006)	0.91
Total spending per prescription	0.992	(0.981, 1.003)	0.15		1.001	(0.993, 1.009)	0.82
**Quarterly health care resource use**							
Total ED visits	1.004	(0.966, 1.044)	0.85		1.009	(0.911, 1.117)	0.87
Total ED spending (USD)	1.019	(0.990, 1.049)	0.21		1.053	(0.988, 1.122)	0.12
Any IP visit	1.050	(0.982, 1.123)	0.15		1.085	(0.946, 1.244)	0.24
Total IP length of stay	1.044	(0.971, 1.121)	0.24		0.929	(0.832, 1.037)	0.19
Total IP spending (USD)	0.975	(0.845, 1.124)	0.72		0.922	(0.830, 1.024)	0.13
Total (ED, IP, prescription) spending (USD)	0.985	(0.924, 1.050)	0.65		1.013	(0.978, 1.050)	0.47

Abbreviations: OR=odds ratio; IRR =incidence rate ratio; CI = confidence interval; ATC = Anatomic Therapeutic Chemical; IP = inpatient; ED = emergency department; USD = 2020 United States dollars.

Source: Author's analysis of Medicaid Analytic eXtract (MAX) claim data (2007-2012).

Notes: Regressions adjusted for covariates listed in methods. Estimates were calculated among all individuals with disabilities in drug cap states (n=8,214) compared with individuals living in comparison states (n=19,832) and the subgroup of individuals with a serious mental illness in drug cap states (n=1,178) and comparison states (n=2,957). Prescription drug outcomes were measured among all individuals on a monthly basis while health care resource use was measured on a quarterly basis in the 12 calendar months before the individual turned age 21. Total spending includes all spending on prescription drugs as well as inpatient and emergency department visits. All results are from the coefficient on the interaction between treated indicator variable and continuous measure of months until 21st birthday in the pre-policy period. All results are reported as incidence rate ratios (IRR) from the zero-inflated negative binomial models except odds ratios (OR) are reported for outcomes from the logistic models (any emergency or any inpatient visit).

© 2021 Geiger CK et al. *JAMA Health Forum.*
